# Mechanisms underlying selecting objects for action

**DOI:** 10.3389/fnhum.2015.00199

**Published:** 2015-04-22

**Authors:** Melanie Wulff, Rosanna Laverick, Glyn W. Humphreys, Alan M. Wing, Pia Rotshtein

**Affiliations:** ^1^School of Psychology, University of BirminghamBirmingham, UK; ^2^Department of Experimental Psychology, University of OxfordOxford, UK

**Keywords:** attention, dual route, semantic interference, action knowledge, conceptual knowledge

## Abstract

We assessed the factors which affect the selection of objects for action, focusing on the role of action knowledge and its modulation by distracters. Fourteen neuropsychological patients and 10 healthy aged-matched controls selected pairs of objects commonly used together among distracters in two contexts: with real objects and with pictures of the same objects presented sequentially on a computer screen. Across both tasks, semantically related distracters led to slower responses and more errors than unrelated distracters and the object actively used for action was selected prior to the object that would be passively held during the action. We identified a sub-group of patients (*N* = 6) whose accuracy was 2SDs below the controls performances in the real object task. Interestingly, these impaired patients were more affected by the presence of unrelated distracters during both tasks than intact patients and healthy controls. Note that the impaired patients had lesions to left parietal, right anterior temporal and bilateral pre-motor regions. We conclude that: (1) motor procedures guide object selection for action, (2) semantic knowledge affects action-based selection, (3) impaired action decision making is associated with the inability to ignore distracting information and (4) lesions to either the dorsal or ventral visual stream can lead to deficits in making action decisions. Overall, the data indicate that impairments in everyday tasks can be evaluated using a simulated computer task. The implications for rehabilitation are discussed.

## Introduction

Activities of daily living such as making a cup of tea are computationally complex and potentially rely on the integration of different cognitive processes (Hartmann et al., 2005). Most of these activities involve interaction with objects and require knowledge on how these objects can be used. Compelling evidence suggests that, perhaps in addition to stored knowledge of object use, an important property of the object is affordance (Gibson, 1979). Objects afford an action through their visual properties such as size, shape, and weight. Furthermore, affordance perception is affected by the context an object is presented in. For example, the pair knife and fork will afford a cutting action while a knife and butter will afford a spreading action. In the case of interacting objects, it is unclear whether potential actions are based only on the visual property of the objects themselves and the procedural/action-based knowledge of how to use the objects together or whether these actions rely on prior conceptual knowledge on how different objects can be used together (cutting vs. spreading). The aim of this study was to investigate decisions about actions with interacting objects, using evidence on impaired actions in neuropsychological patients.

Riddoch et al. (1989) proposed a dual route framework for the retrieval of action knowledge. According to this account, action knowledge can be retrieved either through a direct (non-semantic) route from vision-to-action based on the visual properties of objects (i.e., affordances) or through an indirect (semantic) route by accessing semantic memory. The indirect route is associated with the ventral visual stream representing object knowledge, whereas the direct route is associated with the dorsal visual stream focusing on object use (Ungerleider and Mishkin, 1982; Ungerleider and Haxby, 1994; Milner and Goodale, 1995).

There is some evidence for a double dissociation between the direct and the indirect route, supporting this dual route account. Yoon et al. (2005) reported that a patient with left occipito-temporal brain damage was able to make actions toward objects, even though his object naming and semantic retrieval was impaired (cf. Riddoch and Humphreys, 1987; Hodges et al., 1999). This indicates that prior object recognition is not necessary in order to use objects. In contrast, patients with lesion to the left parietal cortex were not able to act upon objects despite having intact semantic knowledge (Riddoch et al., 1989), implying that preserved access to semantic knowledge is not sufficient for action execution (see Yoon et al., 2002, for a simulation of these results). On the other hand, Tsagkaridis et al. (2014) compared the processing of pairs of objects in healthy controls, patients with temporo-parietal lesions and patients with frontal lesions. Participants had to make a choice in an object triad, selecting the object that had the strongest association with a target object. The object could either form a functional relationship with the target (action-related) or not (non-action-related). All three groups chose action-related pairs over non-action-related pairs, suggesting that action relatedness forms stronger associations between objects than non-action associations. Nevertheless, it was shown that temporo-parietal patients' choices were less affected by action relatedness, compared to the other two groups.

Recent evidence suggests that object use depends on access to semantic knowledge (Hodges et al., 2000; Silveri and Ciccarelli, 2009). Hodges et al. (2000) used the same set of objects to test semantic and action knowledge in semantic dementia patients. The authors showed that, when a patient failed to name an object, he/she was also not able to use it. This indicates a strong link between semantic and action knowledge for object use (cf. Frey, 2007; for neuroimaging evidence, see Chao and Martin, 2000). However, several other studies have demonstrated that patients with parietal lesions were able to name more objects when these were positioned in a way that afforded an action (Riddoch et al., 2003; Humphreys et al., 2010; Wulff and Humphreys, 2013), suggesting some impact of the direct route on naming. Furthermore, Sunderland et al. (2013) showed that patients with ideamotor apraxia were impaired when reaching for a familiar tool but not when reaching for an abstract object. The authors proposed that the selective impairment in tool use is due to a failure to integrate conceptual knowledge (ventral route) to the preserved dorsal route (action knowledge). Evidence for an interaction between semantic and action processing has also been reported with healthy participants (Green and Hummel, 2006; Roberts and Humphreys, 2011; Borghi et al., 2012). For example, Green and Hummel (2006) used a conceptual matching task which required participants to compare a word probe with a target object, separated by a distracter object. Performance was greatest when there was a functional relation between the distracter and target, but only when the distracter was also semantically related to the probe. The results showed that both semantic and action knowledge contributed to the improved performance.

The involvement of the direct and indirect routes in processing objects appears to depend upon task demands and the type of information in the stimuli. Specifically, the way the objects are gripped affects action decisions and neural responses. Mizelle et al. (2013) compared the neuronal responses for correct (e.g., a hammer was congruently gripped and faced toward the nail) and incorrect (e.g., a hammer was incongruently gripped and faced away from the nail) tool use. There was bilateral activity in temporal cortices and insula for incorrect over correct tool use, suggesting that the ventral stream might play a key role in evaluating hand-object interactions, and thus linking action and semantic knowledge. Furthermore, action decisions as opposed to semantic decisions were facilitated for correctly gripped objects than incorrectly gripped objects (Yoon and Humphreys, 2005; Borghi et al., 2012), when the object handle was facing toward the participants (vs. away from them; Yoon and Humphreys, 2007), and when the object position matched the preferred hand position of the participant (Yoon et al., 2010; see Humphreys et al., 2010, for a similar result with neuropsychological patients). Note that both hand grip and object orientation were task-irrelevant in all studies and irrelevant to access to conceptual knowledge, but highly relevant to the direct route to action.

Additionally, an important action cue for interacting objects is the functional role of each object within a pair. Typically in a given action using two objects, one object is used actively (normally using the dominant hand; e.g., pouring with a jug) and the other is held passively (a glass, being poured in to). It has been shown that whether an object is active or passive in the action modulates perception and attention (Riddoch et al., 2003; Roberts and Humphreys, 2010; Wulff and Humphreys, 2013). For example, Roberts and Humphreys (2010) reported that, in temporal order judgments, participants tend to think that the active object is presented prior to the passive one even when the objects appear simultaneously—consistent with the active object being attended first and gaining “prior entry.” In addition, patients showing visual extinction can be biased to report the active member of the pair rather than the passive member, when the items are briefly presented and this occurs even when the active member is on the contralesional (impaired) side (Riddoch et al., 2003; Wulff and Humphreys, 2013).

The interaction between semantic and action knowledge has been investigated using action decisions with object pairs (Laverick et al., 2015). Laverick et al. (2015) reported two experiments that examined the influence of distracters and action cues on tasks requiring the selection of pairs of objects. The study specifically explored the effects of distracters with real objects (Experiment 1) and whether these effects can be replicated with static photographs of the same objects used in the real objects task (Experiment 2). In Experiment 1, participants had to select two objects that can interact with each other among semantically related and unrelated distracters. In Experiment 2, each object was presented as a picture on the screen. Participants judged whether two objects that appear consecutively form a functional action pair. We manipulated the way the objects were gripped and also the order the active and passive objects were presented. We found that action cues and procedural action knowledge guided action decisions. In Experiment 1, the active object was selected before the passive objects with the right (dominant) hand. In Experiment 2, when the active preceded the passive object's picture action decisions were facilitated but this only occurred when the objects were congruently gripped (e.g., Riddoch et al., 2003; Roberts and Humphreys, 2010). With real objects, the semantic relation between the targets and distracters did not affect selecting objects for action. With pictures, on the other hand, action relations were more difficult to reject when two consecutive objects were semantically related (Borghi et al., 2012).

The present study adapted the two experiments from Laverick et al. (2015) in order to examine possible mechanisms underlying selecting objects for action. Specifically, we tested which factors affected the ability of neuropsychological patients to correctly select object pairs for action. Based on the dual framework model (Riddoch et al., 1989), we expected that lesions to temporal, parietal or both cortices would lead to deficits in making action decisions. Based on our previous study (Laverick et al., 2015), we predicted that performances with real objects will be mirrored when a computerized version of the task was used. We assumed that patients who have difficulties with real objects would also show similar deficits when tested on a computer task (and vice versa). If this is the case, this would increase the validity of a computer simulated environment for both assessment and (subsequently) for rehabilitation.

## Experiment 1: selecting real objects for actions

In Experiment 1, we investigated which factors affected the ability to select real objects for action. We asked the following three questions: (i) does the presence of semantically related or unrelated distracters affect paired-object selection?, (ii) does knowledge of the functional role of each object (active/passive) in a pair facilitate object selection?, and (iii) which factors affect patients who fail to select action pairs correctly?. The procedure was adapted from Laverick et al. (2015). The main differences were that the participant could use only one hand and selection was done by touching/pointing the object. The objects were arranged in a semi circle within a reaching distance.

### Methods

#### Participants

A total of 24 participants were recruited from the volunteer panel at the School of Psychology, University of Birmingham. Fourteen neuropsychological patients (one female, *M* = 65.21, *SD* = 8.82, age range: 51–80 years) took part. All except two patients (UB23, UB67) were right-handed [see Table 1, for demographics and clinical data, and Table 2, for Birmingham Cognitive Screen (BCoS; Humphreys et al., 2012) test scores]. In addition, 10 right-handed age-matched healthy volunteers (4 females, *M* = 76.10, *SD* = 6.54, age range: 63–85 years) participated. None of the controls reported any neurological or psychiatric impairment. All participants reported normal or corrected-to-normal vision. Informed consent was obtained from all participants and the study was approved by the local Ethical Review Committee.

**Table 1:** **Demographic and clinical data of the patients**.

**Patient**	**Sex/age/handedness**	**Aetiology**	**Lesion side**	**Time since lesion (years)**	**Lesion volume**	**Patient group**
UB03	M/ 58/ R	Herpes simplex encephalitis (anterior temporal)	B	>15	1,25,441	Impaired
UB10	F/ 62/ R	Stroke (MCA)	B	>15	97,379	Impaired
UB13	M/ 59/ R	Anoxic brain damage (subcortical and parietal)	L	>15	66,911	Intact
UB23	M/ 64/ L	Stroke (MCA)	R	>15	1,41,771	Impaired
UB34	M/ 80/ R	Stroke (CAT and PCA)	R	5	37,499	Intact
UB36	M/ 57/ R	Stroke (PCA)	R	2	89,366	Intact
UB38	M/ 51/ R	Stroke (PCA)	L	5	62,679	Impaired
UB42	M/ 78/ R	Stroke (MCA)	R	5	1,32,949	Intact
UB49	M/ 77/ R	Stroke (subcortical)	L	4	46,738	Intact
UB50	M/ 71/ R	Stroke (PCA)	R	5	54,006	Impaired
UB51	M/ 71/ R	Stroke (MCA)	B	5	80,316	Impaired
UB67	M/ 64/ L	Stroke (subcortical)	B	2	53,821	Intact
UB68	M/ 62/ R	Stroke (subcortical)	B	2	70,471	Intact
UB75	M/ 59/ R	Stroke (MCA)	R	2	1,59,520	Intact

M, male; F, female; R, right; L, left; B, bilateral; MCA, Middle Cerebral Artery; PCA, Posterior Cerebral Artery; CAT, Choroidal Artery Territory.

**Table 2:** **Birmingham Cognitive Screen (BCoS) test scores**.

**BCoS Domain**		**Normative data and cut offs^a^**	**Intact patients (*N* = 8)**	**Impaired patients (*N* = 6)**
Attention	Auditory attention			
	*Accuracy*	46	45.75 (11.70), 3	49.80 (5.31), 2
	*Sustained attention*	>2	0.63 (2.26), 1	1.60 (3.05), 1
	Apple cancelation			
	*Accuracy*	42	39.50 (9.74), 3	40.83 (12.67), 2
	*Asymmetry—full*	< −2 or > 3	3.75 (4.53), 3	2.50 (5.21), 1
Praxis	Multi-step object use	10	10.50 (1.60), 3	11.17 (1.33), 1
	Gesture imitation	9	9.50 (2.14), 4	9.00 (3.74), 2
	Gesture production	9	11.00 (1.31), 2	9.83 (2.93), 1
	Figure copy	37	36.25 (8.24), 2	33.20 (14.02), 3
Language	Picture naming	10	12.63 (1.69), 1	8.17 (6.08), 3
Memory (orientation)	Personal^*^	8	8 (0), 0	6.83 (1.33), 3
	Time and space	6	5.50 (0.76), 3	4.83 (0.98), 4

Standard deviation in parenthesis followed by the number of patients who failed the subtest.

a Normative cut off scores based on 5th percentile age >75 are provided by BCoS (Humphreys et al., 2012).

* A trend for a significant difference between impaired and intact patients was observed for recalling personal information.

#### Apparatus and stimuli

**Figure 1:**
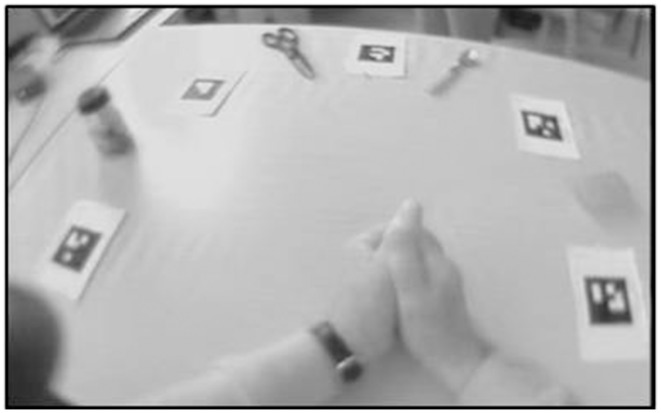
**Example of the trial layout used in the Real object task**. On trial 25, a matching pair (coffee jar and tea spoon) was presented along with two semantically unrelated distracter objects (scissors and soap). The black-and-white sheet of papers represents eye tracking markers needed as reference points for the eye tracking system.

Object selection was carried out with real life objects placed on a table (Figure 1). Thirty-two objects commonly found in the kitchen, office or bathroom, were paired into 16 object pairs that were commonly used together (e.g., mug and tea spoon, see Appendix A in Supplementary Material).

Prior to the experiment, a pilot study was run with a separate group of 20 participants (16 females, *M* = 26.80, *SD* = 8.16). The aim of this pilot study was to evaluate each object pair for action familiarity to include only pairs that were highly familiar. Previous research has shown that unfamiliar pairs compared to familiar pairs require increased object processing demands (Vingerhoets, 2008). For each object pair, participants rated: “How likely are these objects used together? (1-5, 1 = highly unlikely, 5 = highly likely).” In addition, participants indicated for each pair which object was the active object, based on the following definition: “The active object is the one that must be moved in order to perform the action (e.g., paintbrush), whereas the passive object must be held still (e.g., paint pot).” Based on this pilot study, two object pairs (soap-flannel; pencil-sharpener) were excluded as there was less than 60% agreement across participants on the active-passive role within these pairs. Note that the individual objects were still used as distracter objects (see below). Thus, 14 object pairs were used in the present experiment. The mean action familiarity rating was 3.93 (*SD* = 0.97). The ratings for action familiarity and object classification are presented in Appendix A in Supplementary Material.

Performance was video recorded from participants' first-person perspective using the Dikablis eye tracking system (http://www.ergoneers.com/en/hardware/eye-tracking/#dikablis). The system consists of a headband with one camera directed toward the environment that record the participants view and another camera directed toward the left eye pupil. This latter one was used to record the beginning of each trial and to monitor whether the participants have closed their eyes while the objects were placed on the table. The data of the eye tracking will be reported in a separate manuscript.

#### Design and procedure

Each object pair was presented three times: without distracters, with two semantically related distracters and with two semantically unrelated distracters. The distracters were objects from the same stimulus pool. Distracters were defined as semantically related when the target and distracter were commonly found in the same environment, while unrelated distracters were objects from a different environment (cf. Borghi et al., 2012). For the action pair mug and tea spoon, for example, the related distracters were wine glass and fork and the unrelated distracter objects were toothpaste and pen.

These three distracter conditions were administered to each participant in the same order: no distracter, unrelated and related distracter conditions. The order was fixed across participants to ensure that the order would not be a confounding factor, as the main interest was the comparison between the different patient groups. Furthermore, the experiment always started with the no distraction condition to ensure patients were familiar with the object pairs and their functionality. There were 42 trials, 14 for each condition.

The position or side of the presented active/passive object was random across trials. However, the arrangement of objects on the table within trials was the same for all participants. All objects were placed in a semi-circle position within the participant's arm reach. The semi-circle arrangement was used to avoid object occlusions for the purpose of the eye tracking measurement (not reported here). The aim of the experiment was to assess conceptual selection in a simulated real-life environment. In real life, objects are typically oriented or positioned randomly (e.g., a knife can be found on the left or right side of a chopping board with the knife handle facing either toward or away from the person). For this reason, we deliberately placed the objects (targets and distracters) at random positions and orientations on the table. Thus, orientation or position information of the objects could not be used as a cue for selecting the target objects. Additionally, the relative location of the distracters to the target objects was random, i.e., in some trials the targets flanked the distracters, while in other trials the distracters flanked the targets or they were interleaved. Accordingly, the placement (position or orientation) of each object was pseudo-randomized across trials but fixed across participants.

Participants sat in front of a table, with their right and left hand placed on the table. Participants had to select by reaching and touching/pointing the two objects from which they believed form a functional pair (i.e., these two objects which would actively be used together to perform an action). Participants were not instructed to return to the starting point after selecting an object. In the case that participants believed that there was no action pair on the table they were instructed to verbally indicate that there were no objects present that afford an action together. When selecting objects, participants used only one hand, which was their preferred hand. The hand restriction was imposed in order to avoid differences between bi-manual and uni-manual responses associated with healthy controls and patients, respectively.

Prior to each trial, participants had to close their eyes while the objects were arranged on the table. The timing and recording of each trial began when they opened their eyes. This was monitored by the eye tracking system.

#### Data analysis

Video analysis was carried out using ELAN 4.4.0 software (http://tla.mpi.nl/tools/tla-tools/elan/). Trials were annotated for selection accuracy and reaction time (RT). As objects had different sizes and were located at different locations from the participant and from each other, we used the initiation of a movement toward a target as RT measure. By using movement initiation rather than reaching time to the target we ensured that RT was not confounded by differences in target location and size, and this RT measure is assumed to reflect more closely the decision process. For correct trials, RT was defined as the initiation time before the smooth direct arm movement that ended by pointing or touching the target object in relation to the beginning of the trial; this was done separately for active and passive objects (cf. Laverick et al., 2015).

To test the reliability of the coding procedure, the data of one healthy control and one patient were independently analyzed by a second rater. The overall inter-rater correlation was high (*r* = 0.97, *p* < 0.001) for RTs (control *r* = 0.996; patient *r* = 0.922, both *p* < 0.001). There was also full agreement on the accuracy scores.

To identify patients who had difficulties selecting the correct objects, we used the healthy control's overall accuracy rate, across the three distracter conditions of the real object task. Patients whose accuracy was two standard deviations (SDs) below the overall controls mean were classified as impaired patients, while patients within the range of 2SDs were classified as intact patients (Table 1). This classification was used throughout all analyses for Experiment 1 and 2 in order to assess the reasons why impaired patients failed to correctly select objects for action.

For statistical analysis of the accuracy and RT data, a repeated-measures ANOVA was adopted using IBM SPSS Statistic 19 for windows software (SPSS Inc., Chicago, IL). Greenhouse-Geisser correction for degrees of freedom was used when assumption of sphericity was not met. Interaction effects were evaluated with paired *t*-tests (*p* ≤ 0.05).

### Results

#### Impaired and intact patients' difficulties in selecting objects for action

In Experiment 1, we found that six out of 14 patients were reliably impaired in their ability to select objects for action (Table 1). The impaired patients' accuracy was below 2SDs of the mean accuracy of the controls. Using the results from the BCoS (Humphreys et al., 2012), we compared the neuro-cognitive profiles of the two groups of patients. We specifically focused on four cognitive functions associated with the dorsal and ventral route: for the dorsal pathway we used measurements of apraxia and spatial and sustained attention and for the ventral route we used the tests of object recognition and naming and patients' orientation (i.e., knowledge of personal details, time and space; see Table 2). In addition, we compared the overlap and differences in their anatomical lesions (see below).

Apraxia is measured in the BCoS using the following tasks: gesture production and imitation, a multi-step object task assessing the ability to assemble a torch and a complex figure task assessing the ability to visually guide hand movement (Bickerton et al., 2012). Visual-spatial attention is measured using the apple cancelation task and is consider a measure of spatial neglect (Bickerton et al., 2011), while sustained attention is the ability to sustain attention over time measured by the auditory attention. The picture naming task was used to assess the patient's ability to name objects. Finally, the patient's general orientation was evaluated with autobiographical memory questions. Independent *t*-tests (Welch's *t*-test corrected in case of unequal variances) revealed that there were no significant differences between the intact and the impaired patient group regarding apraxia, sustained attention, spatial attention and picture naming (Table 2). However, there was a trend toward significance that impaired patients relative to intact patients were slightly poorer at recalling personal details, *t*_(5)_ = 2.15, *p* = 0.084.

#### Lesion identification

We used automated lesion identification based on fuzzy clustering (Seghier et al., 2008). Patients and healthy controls were scanned at the Birmingham University Imaging Centre (BUIC). An anatomical image was acquired for each participant on a 3T Philips Achieva MRI system using an eight-channel phased array SENSE head coil. The T1 scan was acquired using a sagittal T1-weighted sequence (*TE* = 3.8 ms, *TR* = 8.4 ms, voxel size 1 × 1 × 1). The images were than pre-processed using Seghier et al.'s (2008) modified segmentation algorithm based on the unified segmentation procedure implemented in SPM5 (Statistical Parametric Mapping; Welcome Department of Cognitive Neurology, London, UK). The unified segmentation process uses a-priori maps for the three tissue classes: gray matter (GM), white matter (WM), and cerebrospinal fluid (CSF). Seghier et al. (2008) added a fourth tissue class containing all outliner voxels to account for a potential lesion. The normalized and segmented GM and WM images for each patient were spatially smoothed using a Gaussian kernel of 8 mm full-width-at-half-maximum (FWHM). Next the pre-processed GM and WM images for each patient were compared with these of a group of age-matched healthy controls (>20) to identify voxels that are reliably different between controls and patients (i.e., the lesioned tissue) using fuzzy clustering (Seghier et al., 2008). Finally, a binary lesion map for each patient was computed by combining the lesion voxels in the GM and the WM. For each patient, the volume of the lesion (Table 1) was calculated based on the binary lesion map using Matlab 7.9 (The MathWorks, Natick, MA, USA). Both patient groups had comparable lesion volumes [*t*_(12)_ = −0.53, *p* = 0.605].

**Figure 2:**
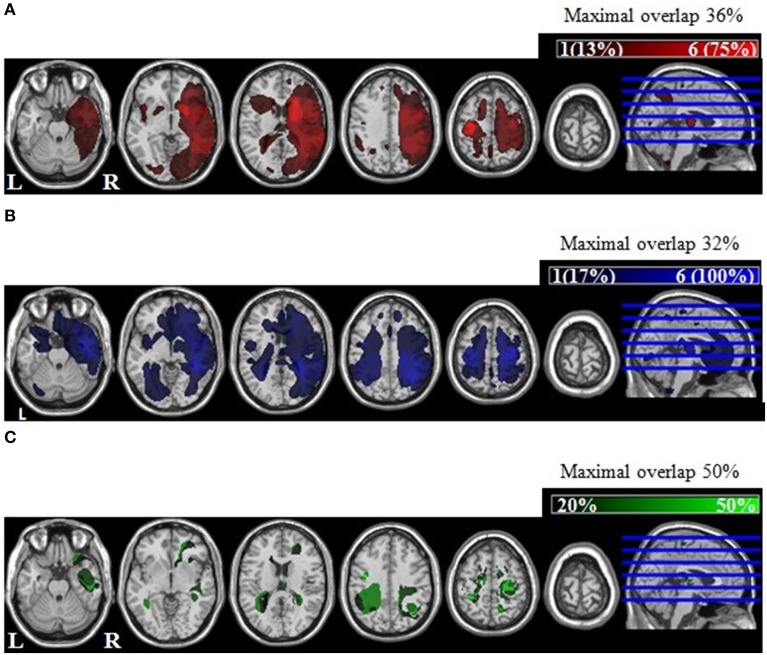
**Lesion reconstructions**. The lesion overlap for **(A)** intact patients (*N* = 8) and **(B)** impaired patients (*N* = 6), scaled from 1 (one patient) to 6 (all patients). **(C)** The lesion difference between impaired and intact patients. The lesions were overlaid on a standard multi-slice template in MRIcron (http://www.sph.sc.edu/comd/rorden/mricron/). Note that the lesion overlap is color coded, with darker color (or %) indicating a higher overlap across patients.

We computed separate overlap maps of lesions for the intact and impaired patient group by adding the binary maps of each patient (Figures 2A,B, respectively). The overlap maps were scaled from 1 to 6 based on number of impaired patients. Accordingly, the lesion overlap map of the impaired patient group shows an overlap between 17% (one patient) and 100% (all six patients; maximal overlap 32%); while for the intact patient group this is equivalent to 13–75% (maximal overlap 36%). To identify regions that were lesioned in the impaired patient group but not in the intact patient group, we first adjusted the lesion overlap maps to account for the different sample size in each group. Then we subtracted the adjusted overlap map of the intact patient group from the adjusted overlap map of the impaired patient group. The lesion subtraction map was scaled for an overlap from 20% (i.e., there was a difference between at least two patients) to 50% to ensure that the lesion difference between the two patient groups was not driven by a single patient. As shown in Figure 2C, impaired patients relative to intact patients had lesions to left parietal, right anterior temporal and bilateral pre-motor regions (see Table 3 for the peak coordinates of the lesion overlap).

**Table 3:** **Anatomical area, MNI coordinates of peak lesion overlap and lesion difference for the comparison intact patients vs. impaired patients (cf. Figure 2C)**.

**Region**	**MNI Coordinate (mm)**			**Δ Lesion overlap**
	*x*	*y*	*z*	
Postcentral gyrus	−32	−38	56	0.50
Precentral gyrus	−34	−30	64	0.50
Precentral gyrus	34	−24	58	0.42
Angular gyrus	41	−62	38	0.41
Angular gyrus	−38	−26	34	0.33
Parahippocampal gyrus	30	4	−32	0.38
Supplementary motor area	−17	−1	63	0.37

#### Performance on the real object task

First, we assessed whether performance was affected by the presence of distracters. Next we investigated whether there was an order difference in selecting the active or the passive member within each object pair. One healthy participant was excluded from all analyses in Experiment 1 due to the repeated use of both hands while performing the task. A summary of the observed effects on action and semantic knowledge for the three groups is presented in Table 4.

**Table 4:**
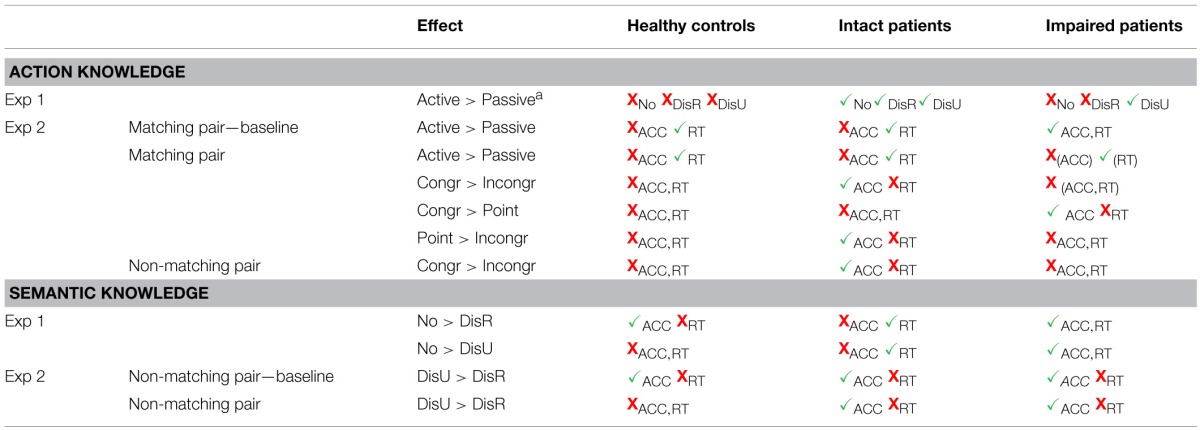
**Summary of the effects on action and semantic knowledge across both experiments**.

Exp 1, Real object task; Exp 2, Computer task; No, No distracter condition; DisR, Related distracter condition; DisU, Unrelated distracter condition; Congr, Congruent hand grip; Incongr, Incongruent hand grip; Point, Pointing hand condition; ACC, Accuracy; RT, Reaction time.

^a^Based on reaction time data.

##### Effects of distracter presence

**Figure 3:**
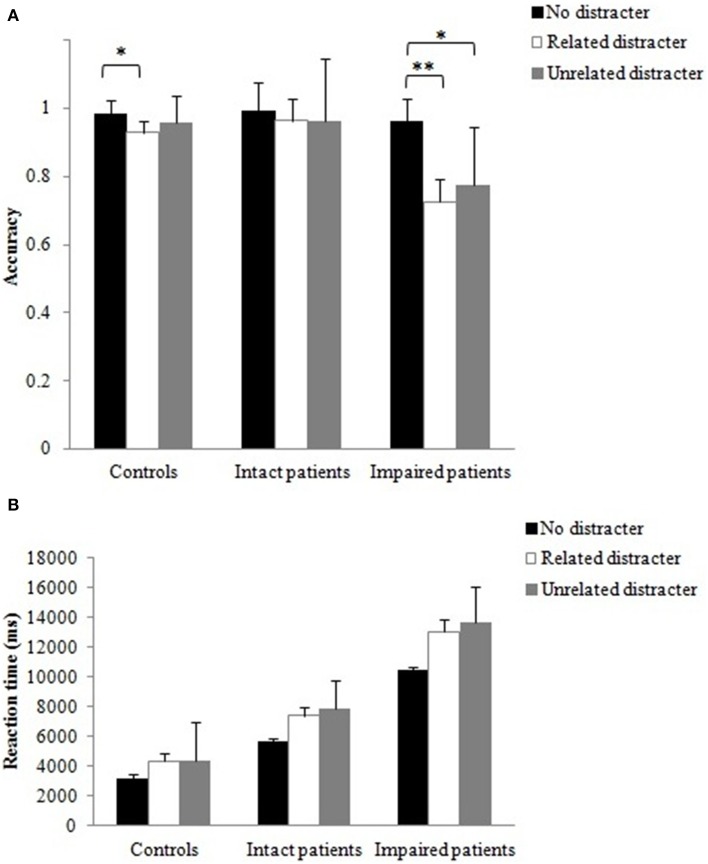
**Real Object task: (A) Mean accuracies and (B) RTs (ms) across distracter conditions with error bars indicating standard error**. Asterisks denote significance (^*^p < 0.05; ^**^p < 0.01).

The accuracy data for each distracter condition were entered into a Two-Way mixed design ANOVA with the within-subject factor distracter (no distracter, related distracter, unrelated distracters) and group (controls, intact patients, impaired patients) as a between-subject factor. Figure 3A shows the mean accuracies for the distracter conditions. The main effect of distracter, F_(2, 40)_ = 15.87, p < 0.001, was significant. Bonferroni corrected multiple comparisons showed that there was a significant decrease in accuracy from the no distracter (0.98) to the related distracter (0.87; p < 0.001) and to the unrelated distracter condition (0.90; p < 0.01), while there was no significant difference between the two distracter conditions. As the groups were chosen based on the accuracy scores, there was obviously a main effect of group, F_(2, 20)_ = 34.57, p < 0.001 (controls (0.96) > impaired patients (0.82), intact patients (0.97) > impaired patients, both p < 0.001; controls = intact patients, p > 0.05).

More interestingly, the distracter by group interaction reached significance, *F*_(3.0, 30.2)_ = 5.49, *p* < 0.01. The three groups did not significantly differ in the no distracter condition. We note that even though the healthy and the intact patient group performed at ceiling one participant in each group indicted that there was no action pair present (i.e., made an error). The impaired patients showed a trend toward significance for more errors than zero in the no distracter condition, *t*_(5)_ = 2.23, *p* = 0.076, with three out of six patients making at least one error. In both distractor conditions, there was a reliable difference between the groups [related distracter: *F*_(2, 22)_ = 40.09, *p* < 0.001; unrelated distracter: *F*_(2, 22)_ = 9.22, *p* < 0.01], with the impaired patients making reliably more errors than intact patient and controls (all *p* < 0.01). We note that though all three groups made reliable more errors than zero in the related distracter condition [controls: *t*_(8)_ = 3.46, *p* < 0.01; intact patients: *t*_(7)_ = 2.64, *p* = 0.033; impaired patients: *t*_(5)_ = 11.78, *p* < 0.001], only controls and impaired patients made reliably more errors in the unrelated distracter condition [controls: *t*_(8)_ = 3.16, *p* < 0.05; intact patients: *t*_(7)_ = 1.80, *p* > 0.05; impaired patients: *t*_(5)_ = 3.30, *p* < 0.05]. Furthermore, we compared the distracter conditions within each group (Figure 3A). For controls and impaired patients, accuracy rate was significantly higher for the no distracter compared to the related distracter condition [controls: *t*_(8)_ = 2.80, *p* < 0.05; impaired patients: *t*_(5)_ = 6.59, *p* < 0.01], while there was a trend toward significance for the intact patient group, *t*_(7)_ = 2.05, *p* = 0.080. For the impaired patients, accuracy rate was significantly higher for the no distracter compared to the unrelated distracter condition, *t*_(5)_ = 2.59, *p* < 0.05, while performance for both controls and intact patients was not affected by the presence of unrelated distracters.

To better understand the result pattern, we investigated in more detail which type of errors each group made. Healthy controls were more likely to indicate that two semantically related objects were also functionally related (average of 1.2 errors) than implying a functional relation between two semantically unrelated objects (average of 0.3 errors). Intact patients showed a similar pattern, implying functional relations between semantically related objects (average of 0.7 errors) but made no errors when there was no semantic relation between the objects. In only one trial (0.14 errors) a patient indicated that he/she cannot identify a functional pair in the search array with semantically related distracters. In contrast, the impaired patient group indicated more often that there was “no functional pair” (average of 3.3 errors), with the effect primarily driven by the unrelated distracter condition (average of 2 errors). Impaired patients also showed more errors of attributing functional relations to semantically related objects (average of 2.28 errors) than to unrelated objects (average of 0.4 errors). Thus, across all participants, semantically related objects were more likely to be assumed to have a functional relation as well.

Taken together, the accuracy data suggest that the impaired patient group showed deficits in selecting functional pairs across all conditions, with the effects being increased when distracters were present. The error analysis indicates that impaired patients had a clear deficit in the knowledge of action relations, as indicated by the frequent failure to identify a functional pair in the search array. Furthermore, impaired patients like intact patients and healthy controls were more likely to attribute action relations to semantically related objects.

##### Effects of active and passive objects on response selection time

RTs for correct trials were entered into a Three-Way mixed design ANOVA with the within-subject factors object role (active, passive), distracter (no distracter, related distracter, unrelated distracter) and group (controls, intact patients, impaired patients) as a between-subject factor. RTs for each distracter condition are presented in Figure 3B. The main effect of distracter, *F*_(1.4, 28.7)_ = 5.78, *p* < 0.05, was significant. Bonferroni corrected multiple comparisons showed that RTs were significantly increased from the no distracter (6456 ms) to the unrelated distracter condition (8647 ms; *p* < 0.01), whereas there was no reliable difference between the related (8277 ms) and unrelated distracter condition and between the no distracter and the related distracter condition. The main effect of group was also reliable, *F*_(2, 20)_ = 21.93 *p* < 0.001. While controls (3985 ms) and intact patients (6985 ms) were significantly faster than impaired patients (12409 ms; *p* < 0.001, *p* < 0.01, respectively), there was a trend toward significance for controls being faster than intact patients (*p* = 0.057). This indicates that the impaired patient group was slower than the intact patient group in selecting objects for action. However, the group by distracter interaction had no effect on selection time.

The object's functional role within a pair affected RTs, *F*_(1, 20)_ = 9.83, *p* < 0.01, with the active object being selected before the passive object. There was a reliable interaction between object role and distracter, *F*_(1.9, 39.5)_ = 5.34, *p* < 0.01. The data showed that the selection priority for the active object was primarily observed when distracters were present. Across participants, the active rather than the passive object was selected first in the unrelated distracter condition, *t*_(22)_ = −3.34, *p* < 0.01, while a trend toward significance was observed for the related distracter condition, *t*_(22)_ = −1.84, *p* = 0.08. There was no significant difference between the selection of the active and passive object in the no distracter condition.

**Figure 4:**
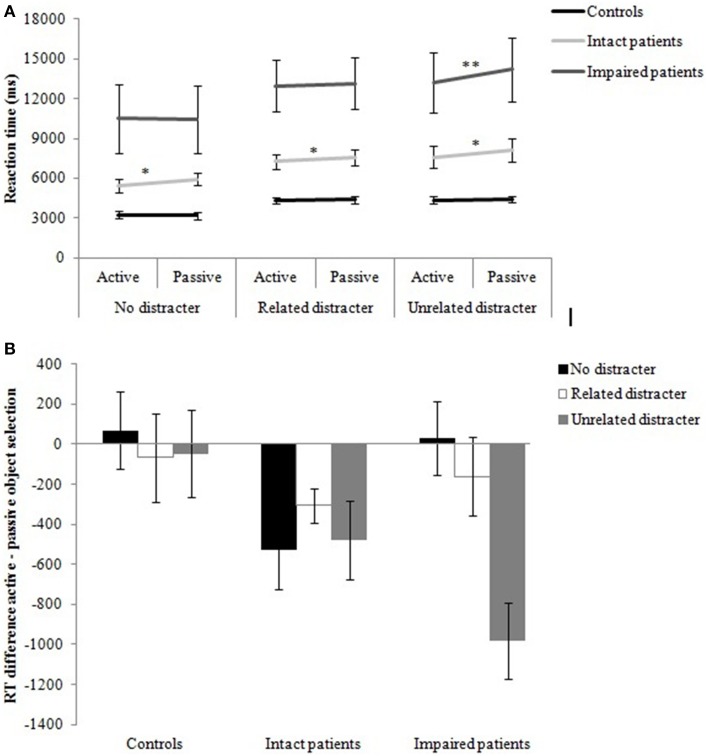
**Real Object task: (A) Mean RTs (ms) for each distracter condition as function of group and whether the active or the passive object was selected first. (B)** RT difference (ms) between the selection of the active and passive object as function of group. Error bars denote standard error. Asterisks denote significance (^*^p < 0.05; ^**^p < 0.01).

Most interestingly, there was a three-way interaction between object role, distracter and group, *F*_(3.9, 39.5)_ = 3.96, *p* < 0.01 (Figure 4A). For all three distracter conditions, intact patients selected the active before the passive object [no distracter: *t*_(7)_ = −2.41; related distracter: *t*_(7)_ = −3.50; unrelated distracter: *t*_(7)_ = −2.45, all *p* < 0.05]. This suggests that intact patients relied on their action procedural knowledge or motor schema when selecting pairs of objects. Impaired patients, reliably prioritized the selection of the active over the passive object only during the unrelated distracter condition, *t*_(5)_ = −5.17, *p* < 0.01. For controls, there was no significant difference between object role and distracter condition. To illustrate the difference between selecting the active and the passive object we computed the time difference for selecting the active and the passive object for each group (Figure 4B). None of the other interactions reached significance.

### Discussion

The results showed that all participants were more accurate and faster when no distracters were present, while all participants were more affected by semantically related distracters relative to unrelated distracters (see below for further details). Similar to the findings reported by Laverick et al. (2015), we observed that the presence of distracters affects paired-object selection. In contrast to our previous study were the semantic relations of distracter showed no reliable effect on selection times (Laverick et al., 2015), here the interference was larger when the distracters were semantically related to the targets, i.e., likely to be found in the same environment (Borghi et al., 2012). We note that here distracter type affected accuracy but not RTs, as there were no reliable RT differences between related and unrelated distracters. The lack of effects on selection time replicates the results reported by Laverick et al. (2015) but not the effects on accuracy. This discrepancy in the results may relate to several methodological differences between both studies. Firstly, the nature of the participants; Laverick et al. tested healthy young and elders as opposed to elders and patients in the present study. Age and neurological condition may have increased reliance on converging information from both action and semantic knowledge when making action decisions. Secondly, the motor restriction on response; in the study of Laverick et al. (2015) participants were allowed the use both hands and they had to demonstrate the action while in the present study participants were instructed to use only one hand and to touch/point the relevant object. The latter response is less associated with an action procedure, and hence may be more susceptible to interference from semantic knowledge. Thirdly, the distracter type was manipulated as an event in the study of Laverick et al. (2015), while in the present study it was manipulated across blocks. Furthermore, the block order was fixed, with no distracter preceding the related distracter and ending with the unrelated distracter condition. Thus, at the unrelated distracter condition participants were most familiar with the target object pairs.

Six out of the 14 tested patients were reliably impaired in selecting objects for action. Interestingly, impaired patients were similarly “distracted” by semantically related and semantically unrelated distracters compared to both healthy controls and intact patients who showed a reliable but weaker interference from related distracters and no interference from unrelated distracters. This implies that any type of object-related distracting information hampered the ability of the impaired patients' to correctly select objects for action. Furthermore, even in the no distracter condition half of the impaired patients failed to identify action relations between objects in at least one trial. We note that all functional pairs were highly familiar, suggesting that some of the impaired patients had deficits in accessing action knowledge. The error pattern of impaired patients also indicates a degraded knowledge of action relations as impaired patients primarily failed to identify a functional pair in the search array (i.e., they frequently indicated that there was no action pair). Moreover, all participants tended to make more pairing errors with semantically related objects, with the effect being increased in the impaired patient group. This indicates that de-selection of inappropriate objects could also play a role in the observed action decision errors.

Another interesting result is that across participants the active object was selected first rather than the passive object of a pair. While previous studies (e.g., Roberts and Humphreys, 2010) could only speculate on the role of action procedural knowledge (i.e., motor schemas) in capturing attention by the active object, here similarly to our previous study (Laverick et al., 2015), we showed with real objects that participants also selected the active object first. More crucially, and in line with our previous study (Laverick et al., 2015), the selection of the active object preceded the passive object primarily when distracters were present, with the effect being increased with semantically unrelated distracters. This suggests that when the layout was more ambiguous, containing multiple affording objects, knowledge of the functional role of each object in a pair as represented in motor schemas facilitated paired-object selection.

We note that active objects were selected first primarily by the patients, with the intact patient group using action procedural knowledge more reliably than the impaired patient group. Interestingly, there was no reliable effect of active vs. passive in the healthy elderly group. This differed from our previous study (Laverick et al., 2015) in which we showed that both young and elders reliably selected the active before the passive object. We suggest that this performance difference relates to the way responses were made, using only one hand in the present study as opposed to both hands in the previous study (Laverick et al., 2015).

The results of the lesion identification analysis showed lesion differences between the intact and impaired patient group. Impaired patients relative to intact patients had lesions to left parietal, right anterior temporal and bilateral pre-motor regions, suggesting that these regions maybe involved in selecting objects for action (see also Mizelle et al., 2013; Sunderland et al., 2013; Tsagkaridis et al., 2014). This also implies that lesions to both the dorsal and the ventral stream led to the impaired patients' poor task performance. Thus, impairments in selecting objects for action can result from lesions to the direct dorsal route or the indirect ventral route (cf. Riddoch et al., 1989). We note that these lesion differences should be treated with caution as they were not statistically significant due to the small sample size.

## Experiment 2: perception of object pairs

In Experiment 1 we used real objects to investigate the ability to select objects for action among distracters. We identified six patients (out of the 14) who showed reliable difficulties in selecting paired-objects for action. The aim of Experiment 2 was to examine whether the observed impairment in paired-object selection also occurred with static photographs of objects (the same objects as those used in the real object task), when presented centrally on a computer screen. Note that in the computer simulated task each object was now presented separately, thus reducing the potential impact of competing distracting items. We presented pairs of objects sequentially, and asked participants to judge whether or not these two objects are commonly used together. To test the effect of the object's functional role within each pair, we manipulated the presentation order of the active and passive object. We predicted that if the selection of an active object precedes the selection of a passive one, then responses would be facilitated for pairs in which the passive object is presented after the active object. Experiment 2 also examined the role of action knowledge in selecting object pairs by manipulating the hand grip presented with the object. Three grip conditions were included: congruent, incongruent, and pointing. In the congruent grip condition the object was gripped appropriately for the familiar functional interaction between two objects. In contrast, even though the incongruent grip was a plausible manipulative grip it did not facilitate the familiar functional interaction between objects. Finally, the role of semantic knowledge in action decisions was examined by looking at the semantic relations between objects that cannot be used together.

### Methods

#### Participants

The same participants as described in Experiment 1 took part in Experiment 2.

#### Apparatus and stimuli

Twenty-six colored photographs of the objects in Experiment 1 were used. In addition, three new objects from the kitchen and office were used. As in Experiment 1, the individual objects were combined into three pairs: (i) matched for action (e.g., pen and paper), (ii) semantically related non-matched for action (e.g., flannel and toothpaste), and (iii) semantically unrelated non-matched for action (e.g., toothbrush and water bottle; see Appendix B in Supplementary Material for non-matching object pairs). The experiment comprised of 13 matched object pairs (Appendix C in Supplementary Material); each object within the pair was classified as either being the active or the passive (see above).

**Figure 5:**
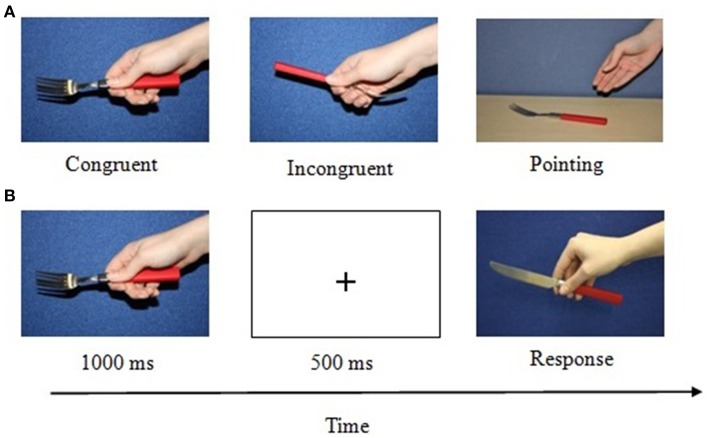
**Computer task: (A) Example stimuli of the different grip conditions. (B)** An example trial sequence for a congruently gripped matching pair. Here the passive object (fork) preceded the active object (knife).

Each object was photographed against a blue background using three grip conditions: congruent grip for action, incongruent grip for action, hand pointing to the object (see Figure 5A). For the grip conditions, pictures were taken from a first-person perspective and objects were gripped with the right hand. For the pointing condition, objects were placed on a table and a right hand pointed to the object. We used only a right hand grasp manipulation to avoid the confounding active-passive manipulation with regard to the holding hand. It has been shown that the hand which holds the objects provides an additional action cue (Humphreys et al., 2010; Wulff and Humphreys, 2013). Finally, as a baseline condition we used a no hand condition. Here the object was photographed without any hand being present; this condition was used to account for the visual effect of hand presence. However, given the difference in the visual complexity of the stimuli between the baseline and the grip conditions we analyzed this condition separately. Note that in the three grip conditions (pointing, incongruent, and congruent grip) visual complexity was not a confounding variable, allowing us to interpret any observed differences based on the way the hand interacted with the object.

#### Design and procedure

The experiment consisted of three grip conditions (congruent, incongruent, pointing) and a baseline control (no hand) condition. The matched pair trials were further divided depending on the object presentation order (i.e., the relation of the present object with the preceding object); the first presented object could be either the active or the passive object (referred to as active 1st or passive 1st, see Appendix C in Supplementary Material). The non-matched pairs were further divided depending on the semantic relation between the objects: semantically related pairs and semantically unrelated pairs (see Experiment 1 for the definition of semantic relatedness).

The experiment was divided into three runs (240 trials in total). In each run, there were four blocks (no hand, pointing hand, congruent grip, incongruent grip). The order of the blocks was pseudo-random. Within each grip block, 15 object pairs were presented pseudo-randomly but fixed across participants: 10 matching pairs (half of them were presented with the active preceding the passive and vice versa) and five non-matching pairs (three semantically related and two semantically unrelated object pairs). The inclusion of twice as many functional pairs was done to facilitate and prime action knowledge processes.

The stimuli were presented in pairs and participants performed an action decision task on each pair (i.e., is the present stimulus commonly used with the preceding stimulus?). The stimuli were presented in the center of the computer screen on a white background. Each trial began with the presentation of a black central 500 ms fixation cross. Then the first object picture was presented for 1000 ms, followed by a black central 500 ms fixation cross. Next a secondary object picture was presented until the participant's response (Figure 5B). Participants had to respond as quickly and as accurately as possible by pressing a corresponding key on a keyboard using the middle and index finger of their preferred hand. Participants were instructed that their decision should only be based on whether the objects would be used together, and not on contextual relation (such as both objects can be found in the kitchen) or similarity of shape or look. No time limit was set for participants' response. Prior to the experiment, participants were given 10 practice trials; these results were not included in the data analysis.

The stimuli were presented with E-prime software (Version 2.1; Psychology Software Tools, 2006). Visual stimuli were displayed on a 19-inch monitor at a viewing distance of approximately 60 cm. Stimuli viewing size were roughly 8.5 degree.

### Results

The same division of the two patient groups as in Experiment 1 was used. Trials in which RTs were less than 200 ms (*n* = 4) or greater than 10 s (*n* = 25) were removed from the analysis. For five participants, data of one session was lost due to technical problems.

In order to explore the effects of grip across matching and non-matching object pair conditions, separate ANOVAs were performed. The observed effects on action and semantic knowledge for the three groups are presented in Table 4.

#### Matching object pair condition

First of all, we analyzed the data for the baseline (only the object was depicted) to investigate the effect of action relation between objects, without being influenced by the effect of hand presence. The accuracy data for the baseline condition were entered into a Two-Way mixed design ANOVA with the within-subject factors being object presentation (active 1st, passive 1st); group (control, intact patients, impaired patients) was treated as a between-subject factor. The main effect of object presentation, *F*_(1, 21)_ = 5.66, *p* < 0.05, reached significance, with the active object (0.92) being more accurately selected compared to the passive object (0.89). There was also a main effect of group, *F*_(2, 21)_ = 16.44, *p* < 0.001. Bonferroni corrected multiple comparisons showed that controls (0.98) performed significantly better than impaired patients (0.77; *p* < 0.001), intact patients (0.96) performed better than impaired patients (*p* < 0.001), while there was no reliable difference between controls and intact patients. There was a reliable interaction between object presentation and group, *F*_(2, 21)_ = 16.29, *p* < 0.001. Impaired patients were more accurate when the active object rather than the passive object appeared first, *t*_(5)_ = 4.28, *p* < 0.01, while the opposite result was observed for the intact patients, *t*_(5)_ = 2.46, *p* < 0.05. In contrast, the accuracy of the control participants' was not affected by object presentation.

The same analysis was conducted for the RT data. The main effect of object presentation was reliable, *F*_(1, 21)_ = 13.33, *p* < 0.01, with faster responses when the active (1283 ms) preceded the passive object (1427 ms). There was also a significant main effect of group, *F*_(2, 21)_ = 13.87, *p* < 0.01. Bonferroni corrected multiple comparisons showed that controls (846 ms) were significantly faster than impaired patients (2004 ms; *p* < 0.001), intact patients (1215 ms) performed better than impaired patients (*p* < 0.01), whereas there was no reliable difference between controls and intact patients.

The results for the baseline (no hand) condition highlighted that action procedural knowledge facilitated action decisions; thus for impaired patients action decisions were more accurate when an active object preceded the passive object. As in our previous study (Laverick et al., 2015), this effect was not observed with healthy controls. Surprisingly, the effect was reversed in the intact group. Intact patients appeared to show a trade-off effect between accuracy and RT. They responded faster when the active object preceded the passive object, but they also made more errors selecting the active over the passive object. This may suggest that the active object increased motor excitability, leading to rushed responses in the price of less accuracy.

**Figure 6:**
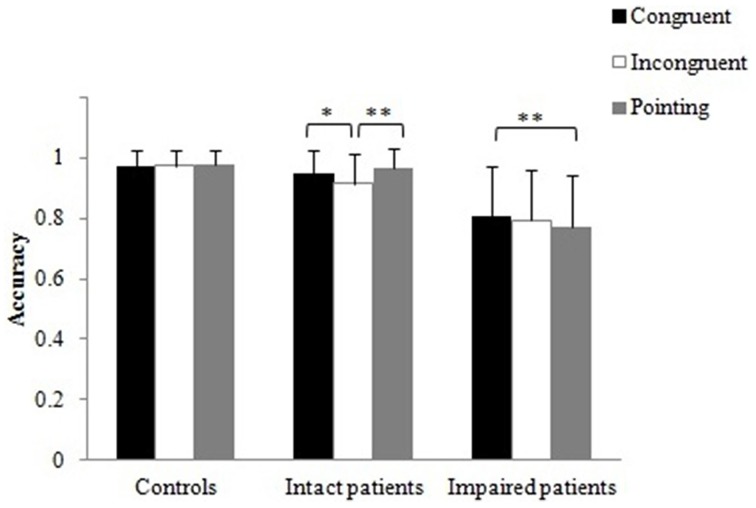
**Computer task: Mean accuracies for matching object pair conditions as function of grip type**. Error bars denote standard error. Asterisks denote significance (^*^p < 0.05; ^**^p < 0.01).

The accuracy data for the three grip conditions were entered into a Three-Way mixed design ANOVA with the within-subject factors being grip (congruent, incongruent, pointing) and object presentation (active 1st, passive 1st); group (control, intact patients, impaired patients) was treated as a between-subject factor. The main effects of grip and object presentation did not reach significance. There was a main effect of group, *F*_(2, 21)_ = 7.35, *p* < 0.01. Bonferroni corrected multiple comparisons showed that controls (0.98) performed significantly better than impaired patients (0.80; *p* < 0.01), intact patients (0.94) performed better than impaired patients (*p* < 0.05), whereas there was no reliable difference between controls and intact patients. This confirms that patients who had difficulties in selecting real objects for action (Experiment 1) also had difficulties when making action decisions on pictures presented one at a time. The grip by group interaction was significant, *F*_(3.6, 37.8)_ = 3.34, *p* < 0.05 (Figure 6). Intact patients benefited from the congruent grip condition, showing increased accuracy in the congruent compared to the incongruent grip condition, *t*_(7)_ = 3.00, *p* < 0.05, and accuracy was also higher for the hand pointing compared to the incongruent grip condition, *t*_(7)_ = 4.46, *p* < 0.01. This suggests that the incongruent grip interfered with the action decision task. In contrast, impaired patients' accuracy was only significantly increased when the grip was congruent relative to the hand pointing condition, *t*_(5)_ = 7.06, *p* < 0.01; there were no reliable differences between congruent and incongruent grip conditions. Notably, accuracy for healthy controls' was not affected by the grip manipulation. No other interactions were significant.

RTs. The main effect of object presentation was reliable, *F*_(1, 21)_ = 8.74, *p* < 0.01, with faster responses when the active rather than the passive object appeared first. There was also a significant main effect of group, *F*_(2, 21)_ = 14.88, *p* < 0.001. Bonferroni corrected multiple comparisons showed that impaired patients (2095 ms) were significantly slower than controls (868 ms; *p* < 0.001) and intact patients (1207 ms; *p* < 0.01), and that RTs did not reliably differ between controls and intact patients. This suggests that impaired patients were not only less accurate but also needed longer to make an action decision. No other main effects or interactions were significant.

#### Non-matching object pair conditions

As before, we first analyzed the data for the baseline to investigate the effect of semantic relatedness between objects, without being influenced by the effect of hand grip. The accuracy data for the baseline condition were examined with a Two-Way mixed ANOVA with the type of the semantic relation between the object pair (related, unrelated) as the within-subject factors; group (control, intact patients, impaired patients) was treated as the between-subject factor. There was only a significant main effect of semantic relation, *F*_(1, 21)_ = 7.29, *p* < 0.05, with performance being more accurate when unrelated (0.92) than related distracters (0.84) were present. There was a trend toward significance for a main effect of group, *F*_(2, 21)_ = 2.776, *p* = 0.085 (controls = 0.95, intact patients = 0.90, impaired patients = 0.79). No other interactions were significant.

RTs. There was only a significant main effect of group, *F*_(2,21)_ = 11.75, *p* < 0.001; controls (1037 ms) > impaired patients (2609 ms), *p* < 0.001; intact patients (1679 ms) > impaired patients, *p* < 0.05; controls = intact patients, *p* > 0.05. No other main effects or interactions were significant.

**Figure 7:**
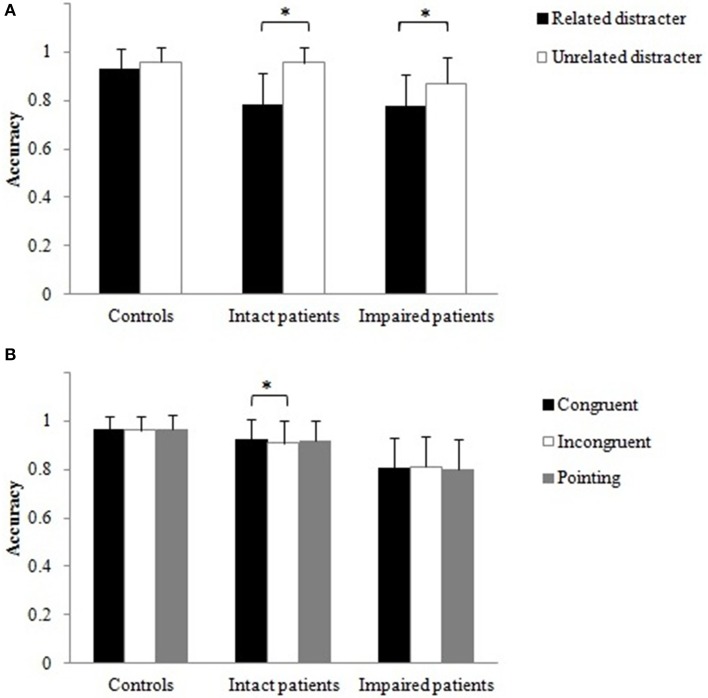
**Computer task: Mean accuracies (with standard error) for non-matching object pair conditions**. The data were pooled across **(A)** distracter and **(B)** grip conditions. Asterisks denote significance (^*^p < 0.05).

The accuracy data for the three grip conditions were examined with a 2 × 3 × 3 [semantic relation between the object pair (related, unrelated) × grip (congruent, incongruent, pointing) × group (control, intact patients, impaired patients)] ANOVA. There was only a significant main effect of semantic relation, *F*_(1,21)_ = 14.26, *p* < 0.01. Similar to Experiment 1 with real objects, accuracy was higher when objects were semantically unrelated (0.94) compared to when they were semantically related (0.84). There was a trend toward significance for a semantic relation by group interaction, *F*_(2, 21)_ = 2.84, *p* = 0.081 (Figure 7A); the effect of semantic relation was reliable for both patient groups, with intact and impaired patients being less accurate when the objects were semantically related compared to when they were unrelated, *t*_(7)_ = 2.56, *t*_(5)_ = 2.99, both *p* < 0.05, respectively. In contrast, healthy controls were unaffected by the semantic relation between the stimuli. There was also a trend for a reliable grip by group interaction, *F*_(3.7, 39.3)_ = 2.60, *p* = 0.054 (Figure 7B). Here, the intact patients benefited when the grip was congruent compared to when the grip was incongruent, *t*_(7)_ = 2.43, *p* < 0.05, but this additional action information was not used by the impaired patients and healthy controls. None of the other main effects or interactions reached significance.

**Figure 8:**
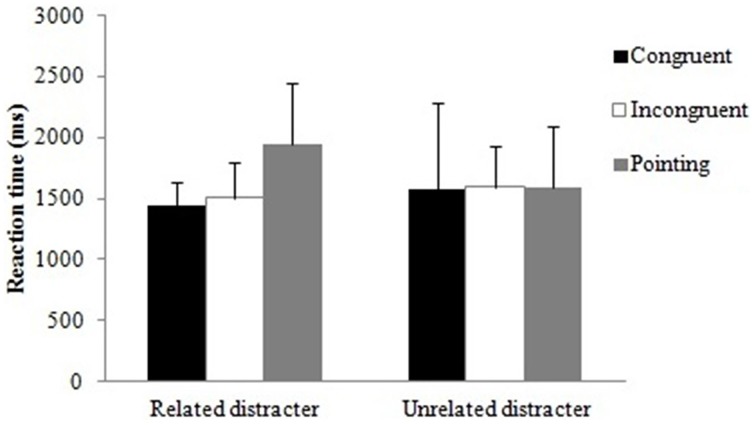
**Computer task: The interaction between distracter and grip for non-matching object pair conditions**. Error bars denote standard error.

RTs. There was a significant main effect of group, *F*_(2, 21)_ = 7.25, *p* < 0.01. Bonferroni corrected multiple comparisons showed that controls (1070 ms) were significantly faster than impaired patients (2555 ms; *p* < 0.01), whereas there were no significant differences in RT between controls and intact (1659 ms) patients and between intact and impaired patients. The semantic relation by grip interaction was significant, *F*_(1.4, 30.3)_ = 5.12, *p* < 0.05. There was a trend toward significance that participants were slower rejecting an action relation between objects when the objects were semantically related in the context of a pointing hand compared to when these objects were congruently gripped, *t*_(23)_ = 1.96, *p* = 0.063 (Figure 8). No other main effects or interactions were significant.

### Discussion

Across all participants we replicated our previous results (Laverick et al., 2015) showing effects of semantic relation and hand grip on action decisions. In the absence of hand information, impaired patients showed reliably poorer performance compared to intact patients and healthy controls. Moreover, across participants, action decisions were facilitated when the active object preceded the passive object. Finally, semantic relations interfered with action decisions, making it more difficult to reject action relations between semantically related objects.

In the three grip conditions and when objects were matched for action, action decisions were facilitated when the active object was presented before the passive object. Both intact and impaired patients made fewer errors when the objects were congruently grasped compared to when the hand grip was incongruent or when a pointing hand was presented. Note that the incongruent grip interfered with action decisions made by intact patients but not these made by impaired patients. Performance of healthy controls' was not affected by the semantic relation between the objects, presumably due to ceiling effects.

When objects were not matched for action and presented without a hand grip, there was better performance with semantically unrelated objects than with related objects across all participants. This replicates our previous results (Laverick et al., 2015) and confirms the results of Experiment 1, suggesting that semantic information had a disruptive effect on performance across both reported experiments. This implies that semantic information affects paired-object selection, especially in the absence of action information. As in Experiment 1, impaired patients were affected by semantic information. However, impaired patients were less affected by the presence of semantic information when making action decisions with real objects, but they were more affected when action cues were weak, i.e., when objects were presented as pictures on a computer screen.

There was also an effect of object presentation for matched pairs across all participants. Responses were faster when the active rather than the passive object appeared first, replicating the results with real objects and confirming previous results with healthy participants (Roberts and Humphreys, 2010) and neuropsychological patients (e.g., Riddoch et al., 2003; Wulff and Humphreys, 2013).

## General discussion

Our study investigated which factors affected paired-object selection in neuropsychological patients and healthy controls by manipulating the nature of the task: using real objects (Experiment 1) and presenting pictures of objects on a computer screen (Experiment 2). The presence of semantic information had a disruptive effect on paired-object selection across both experiments while the presence of action information boosted performance. We first demonstrated similar performance across two different experimental set-ups (selecting real objects vs. responding to pictures of objects) in healthy controls and neuropsychological patients.

Interestingly, despite the differences between Experiment 1 and 2 with respect to the format of the object presentation (real objects vs. pictures on a computer screen, multiple objects vs. single objects) and the required response (touching/pointing to an object vs. pressing a keyboard) a similar pattern of impairments was observed. Most interestingly, impaired patients (defined based on their interaction with real objects) also showed the lowest performance level (RTs and errors) when making action decisions on stimuli presented on a computer screen. Taken together, the data indicate that action knowledge can be estimated using a computer task, providing a good simulation for interacting with real objects in daily living.

Healthy controls and intact participants performed well in the real object task when no distracters were present, while performance was more affected by semantically related distracters compared to unrelated distracters. More crucially, impaired patients made a similar amount of errors with related and unrelated distracters, and also made numerically more errors in the no distracter condition. This indicates that the impairment in the real object task maybe due to degraded access to both action and semantic knowledge. In comparison with the intact patient group, impaired patients also showed weaker effects of action knowledge and semantic knowledge in the computer task. This is in line with the anecdotal observation of the lesion subtraction analysis: Impaired patients, in contrast to intact patients, had lesions to anterior temporal regions which are associated with semantic processing (for a recent meta-analysis, see Visser et al., 2010; see also Chao et al., 1999; Grill-Spector, 2003), especially the processing of an object's function (e.g., Kellenbach et al., 2005; Canessa et al., 2008). The present results support the involvement of semantic knowledge in action decisions, confirming the semantic route from vision-to-action (Riddoch et al., 1989).

There was also evidence for the involvement of the direct route from vision-to-action across all participants in both experiments, though the effects were magnified in the patient groups (strongest in the intact patient group) and weakest in the healthy control group. Across both experiments, we found a bias toward the active object over the passive object. Note that this occurred even though there was no need to perform any actions with the objects, and the response was made by touching/pointing the objects (Experiment 1) or pressing a response key (Experiment 2). This is in accordance with previous findings, suggesting that attention is captured by the active object first, as shown in computerized temporal order judgment studies with young healthy participants (Roberts and Humphreys, 2010) and in object naming studies with neuropsychological patients (Riddoch et al., 2003; Wulff and Humphreys, 2013). Crucially, the pronounced report of the active object was primarily found when distracters were present (Experiment 1). This drawing of attention to the active object seems to facilitate the identification of the passive object when selection becomes more difficult and when the action is afforded by the context. These data emphasize the importance of action knowledge for everyday activities (cf. Tsagkaridis et al., 2014). The special role of active objects (i.e., tools) has been highlighted in several behavioral and neuroimaging studies where tools elicit a stronger pre-motor and parietal response compared to other objects (for converging EEG data, see e.g., Handy et al., 2003; Handy and Tipper, 2007; Proverbio et al., 2011; Goslin et al., 2012; see also Chao et al., 1999; Chao and Martin, 2000, for fMRI evidence).

Further evidence for the direct route comes from the grip manipulation in Experiment 2. Across both matching and non-matching object pairs, intact patients showed better performance for objects with congruent compared to incongruent grip, whereas the effect of grip was less pronounced in impaired patients. This indicates that impaired patients had degraded access to action knowledge. The effect of hand grip in the intact patient group (congruent > incongruent) fits well with previous data with healthy participants using single objects, suggesting that the visual properties of objects (including how to grasp an object) can improve action decisions (Yoon and Humphreys, 2005; Borghi et al., 2012). However, we failed to find evidence that the hand grip had an effect on performance in healthy controls. It could be that the present task was too easy for healthy participants (as indicated by the high accuracy) leading to ceiling effects. In our previous study (Laverick et al., 2015), the task was more difficult and resulted in reliable effects of grip for both young and healthy elderly controls. We propose that task demands and stimulus information can determinate how strong the direct route is activated.

The present data indicate that impaired patients were able to process at least some types of action information. Impaired patients were able to utilize knowledge on motor procedures as they were biased to select/process the active object before the passive object and benefited from a congruent grip compared to a pointing hand. Similarly, impaired patients appeared to rely on semantic information when making action decisions, but only when objects were presented as pictures on the screen (Experiment 2). Here they showed worse performance for semantically related compared to unrelated distracters. This suggests that selecting objects for action can be impaired following degraded access to both semantic or action knowledge systems. This proposal is in accordance with the identified lesion differences between intact and impaired patients. Impaired patients had lesions to the right anterior temporal cortex, left parietal cortex or bilateral pre-motor areas. Lesions to the ventral route (anterior temporal areas) are typically associated with semantic processing (Visser et al., 2010), while lesions to the dorsal route (parietal cortex, pre-motor areas) are thought to play a key role in visuo-motor processing for goal-directed actions (Goodale and Milner, 1992). More precisely, the posterior parietal cortex is commonly associated with the retrieval of action knowledge (i.e., the appropriate hand and finger movement for tool use), whereas the pre-motor cortex is involved in motor preparation. The present results are consistent with the proposed interaction between action and semantic knowledge. Sunderland et al. (2013), for example, suggested that the impaired tool use in ideamotor apraxia is caused by a failure to integrate conceptual knowledge with action knowledge. Similarly, Mizelle et al. (2013) suggested that conceptual knowledge is necessary for understanding functional relations between objects (“functional affordance”), and thus linking action-conceptual and action-procedural systems.

The present data provide further evidence for the dual route framework, suggesting that paired-object selection is mediated either by a direct route, which is sensitive to action knowledge or by a semantic route which, in turns, depends on access to semantic knowledge. Accordingly, impaired action decision performance can be caused due to lesions to both visual pathways. Identifying the impaired processing route will allow an individualized rehabilitation approach by training either action or semantic knowledge to reduce interference from distracters.

## Study limitations

One of the limitations of the present study is the relative small number of patients in each group. In the present study, we did not recruit patients to groups based on their cognitive profiles and lesion sites before study participation. This was done to ensure that the recruitment strategy would not confound the results. Patients were classified into intact and impaired patients based on their performance in the real object task, resulting in different sample sizes for each patient group. Moreover, both patient groups were heterogeneous in terms of their cognitive profiles and lesion sites. Therefore, a differentiation between lesions to the dorsal and the ventral route was not possible with the present patient sample. However, the data suggest that lesions to either the dorsal or the ventral route can lead to deficits in making action decisions. Future research with allocation of patients based on their lesion location is needed to gain a better understanding of the different contribution of the dorsal and ventral route to action knowledge.

In the present experiment we used a block design for manipulating distractor type in the real object task and grip type in the computer task. Furthermore, we presented the blocks in pseudo-random order but fixed across participants to investigate possible differences across participants. This may have led to a condition order effect. We note that this should be taken into account when interpreting the results.

Variability in the types of functional relations between objects may have also reduced the overall power of the study. Six out of 14 object pairs (e.g., knife and fork; Appendix A in Supplementary Material) afforded a more distal action (e.g., a fork and a knife are used together with a third food substance). This type of interaction is common for most object pairs that can be found in the kitchen, whereas other object pairs did not require a third substance for a meaningful functional interaction (e.g., scissors and paper, toothpaste and toothbrush). It is unclear how the different functional types of action relations impact the observed results. Despite this limitation, it has been found that paired-object selection was affected by semantic and action procedural knowledge, regardless of the type of functional relation. However, further research is needed to clarify whether and to what extent the type of functional relation (direct/indirect) plays a role in making action decision.

There was high agreement across participants on the functional role (active vs. passive) of each object within a pair, however it was not perfect. Furthermore, it is known that the functional role of each object within a pair depends on the way these objects are used and it is affected by culture. For example, in Europe the knife is held with the dominant (right) hand and the fork with the left hand for both eating and cutting. This implies that the knife compared to the fork plays a more active role in European table manners. The functional role of knife and fork is reversed in American table manners for cutting and eating, highlighting that the fork plays a more active role in American cultures (http://www.thekitchn.com/survey-using-your-knife-and-fork-166188). Thus, in future studies it will be informative to classify active and passive roles of objects within a pair based on individual habits.

Finally, the present sample included mostly men. This is because stroke is more common in men than in women (Appelros et al., 2009). It is difficult to know whether the results can be generalized across genders. We believe they can be generalized, given previous literature (e.g., Laverick et al., 2015; Borghi et al., 2012), but this obviously needs to be tested in future studies.

## Conclusion

We showed that paired-object selection is affected by semantic and action procedural knowledge. The data are consistent with the existence of two routes from vision-to-action: a semantic route which is activated when distracters are present and a direct route which is activated when action-related cues are present. Lesions to either the direct or the semantic route can have an impact on action decisions.

### Conflict of interest statement

The authors declare that the research was conducted in the absence of any commercial or financial relationships that could be construed as a potential conflict of interest.
