# Supplementary material for: Mechanisms underlying selecting objects for action
Source: Front Hum Neurosci. 2015 Apr 22;9:199. doi: 10.3389/fnhum.2015.00199 (PMC4406091; doi:10.3389/fnhum.2015.00199)
Supplement: Supplementary file 1 [file DataSheet1.DOCX]

**Appendix A: Stimuli used in the Real Object task**

|  |  | |  | |  |  |
| --- | --- | --- | --- | --- | --- | --- |
| Active (%) | | Passive | | Action familiarity rating (SD) | Semantically related distracters | Semantically  unrelated distracters |
| Pen (1.0) | | Paper | | 5.00 (0) | Sellotape, staples | Soap, knife |
| Toothbrush (0.65) | | Toothpaste | | 4.90 (0.45) | Razor, soap | Pencil, teabag |
| Large spoon (1.0)* | | Soup bowl | | 4.40 (0.75) | Teabag, water bottle | File, pencil |
| Knife (0.8)* | | Fork | | 4.20 (0.89) | Teabag, large spoon | Soap, paper |
| Teaspoon (1.0)* | | Coffee jar | | 4.00 (0.92) | Water bottle, knife | Scissors, soap |
| Scissors (1.0) | | Paper | | 3.90 (0.72) | Staples, sharpener | Knife, teabag |
| Teabag (0.8)* | | Mug | | 3.85 (0.81) | Glass, knife | Plastic wallet, toothpaste |
| Teaspoon (1.0)* | | Mug | | 3.70 (0.66) | Wine glass, fork | Toothpaste, pen |
| Soap (0.65) | | Sponge | | 3.60 (1.14) | Toothbrush, razor | Pen, fork |
| Hole punch (0.95) | | Paper | | 3.40 (0.99) | Sharpener, file | Flannel, mug |
| Pen (1.0) | | Envelope | | 3.40 (0.94) | File, scissors | Coffee jar, razor |
| Rubber (1.0) | | Paper | | 3.20 (0.89) | Sharpener, sellotape | Fork, teabag |
| File (0.6) | | Plastic wallet | | 3.15 (0.88) | Scissors, pen | Soap, toothpaste |
| Water bottle (0.8)* | | Glass | | 2.95 (0.83) | Teabag, fork | Soap, scissors |

*Note.* Ratings range from 1 (highly unlikely to be used together) to 5 (highly likely to be used together).

*These object pairs afford a more distal action, i.e. requiring a third substance (e.g., food).

**Appendix B: Object presentation in the Computer task**

|  |  |  |
| --- | --- | --- |
| Object 1^st^ | Object 2^nd^ | Object pair condition |
| Soap (A) | Sponge (P) | Matching pair |
| Paper (P) | Rubber (A) | Matching pair |
| Toothpaste | Flannel | non-matching pair but semantically related |
| Large Spoon | Fork | non-matching pair but semantically related |
| Large Spoon | Mug | non-matching pair but semantically related |
| Knife | Pen | non-matching pair but semantically unrelated |
| Toothbrush | Water bottle | non-matching pair but semantically unrelated |

*Note.* A = active, P = passive.

**Appendix C: Matching pairs used in the Computer task**

|  |  | |  |
| --- | --- | --- | --- |
| **Active** | | **Passive** | |
| Knife | | Fork | |
| Pen | | Paper | |
| Pen | | Envelope | |
| Rubber | | Paper | |
| Soap | | Sponge | |
| Stapler | | Paper | |
| Staples | | Stapler | |
| Teabag | | Mug | |
| Teaspoon | | Mug | |
| Teaspoon | | Coffee jar | |
| Toothbrush | | Toothpaste | |
| Water bottle | | Glass | |
| Water bottle | | Wine glass | |
